# Patterns of univariate and multivariate plasticity to elevated carbon dioxide in six European populations of *Arabidopsis thaliana*

**DOI:** 10.1002/ece3.5173

**Published:** 2019-05-08

**Authors:** Mark Jonas, Brandon Cioce

**Affiliations:** ^1^ Department of Biology, School of Natural and Social Sciences State University of New York—Purchase College Purchase New York

**Keywords:** *Arabidopsis thaliana*, common principal components analysis, eigenvalue variance, elevated carbon dioxide, global climate change, multivariate phenotypic plasticity, phenotypic integration

## Abstract

The impact of elevated carbon dioxide on plants is a growing concern in evolutionary ecology and global change biology. Characterizing patterns of phenotypic integration and multivariate plasticity to elevated carbon dioxide can provide insights into ecological and evolutionary dynamics in future human‐altered environments. Here, we examined univariate and multivariate responses to carbon enrichment in six functional traits among six European accessions of *Arabidopsis thaliana*. We detected phenotypic plasticity in both univariate and multivariate phenotypes, but did not find significant variation in plasticity (genotype by environment interactions) within or among accessions. Eigenvector, eigenvalue variance, and common principal components analyses showed that elevated carbon dioxide altered patterns of trait covariance, reduced the strength of phenotypic integration, and decreased population‐level differentiation in the multivariate phenotype. Our data suggest that future carbon dioxide conditions may influence evolutionary dynamics in natural populations of *A. thaliana*.

## 1 INTRODUCTION

The impact of future atmospheric carbon dioxide concentrations on plants is a pressing concern in global change biology (Jump & Penuelas, 2005; Leakey & Lau, 2012; Terrer et al., 2017; Ward & Kelly, 2004). The current global concentration of carbon dioxide is above 400 parts per million (ppm), and it is projected to double within a century (IPCC, 2014). A growing body of work focused on univariate plant traits suggests that phenotypic plasticity, or the ability of a genotype to express two or more phenotypes depending on the environment, will be an important short‐term response mechanism to elevated carbon dioxide (Anderson, Inouye, McKinney, Colautti, & Mitchell‐Olds, 2012; Franks, Weber, & Aitken, 2014; Jump & Penuelas, 2005; Nicotra et al., 2010). Studies have shown that carbon enrichment alters plant growth, phenology, and fitness (Van der Kooij, Kok, & Stulen, 1999; Makino & Mae, 1999; Perry, Shafroth, Blumenthal, Morgan, & LeCain, 2013; Ward & Kelly, 2004; Ward & Strain, 1999), and in turn, these responses can influence competitive interactions (Lau, Shaw, Reich, & Tiffin, 2014; Stiling & Cornelissen, 2007), community structure (Stinson & Bazzaz, 2006), ecosystem productivity (Stock & Evans, 2006; Ward & Kelly, 2004), and evolutionary dynamics of plant populations (Collins & Bell, 2004; Lau et al., 2014; Potvin & Tousignant, 1996; Ward, Antonovics, Thomas, & Strain, 2000).

It is widely recognized that phenotypic plasticity involves coordinated changes in multiple traits and that multivariate analyses can uncover aspects of genetic architecture and development that may be challenging to detect with univariate approaches alone (Chirgwin, Monro, Sgro, & Marshall, 2015; Etterson & Shaw, 2001; Teplitsky et al., 2014; Walsh & Blows, 2009). Importantly, variation in genetic architecture and development may influence both short‐term plastic responses and long‐term adaptation to rapidly changing environments (Chirgwin et al., 2015; Conner, Cooper, Rosa, Pérez, & Royer, 2014; Etterson & Shaw, 2001; Forsman, 2015; Hellmann & Pineda‐Krch, 2007; Lind, Yarlett, Reger, Carter, & Beckerman, 2015; Murren, 2012; Pigliucci & Preston, 2004; Teplitsky et al., 2014; Walsh & Blows, 2009). Since one of the primary aims of global change biology is to predict short‐ and long‐term responses to rapid climate change, this idea has prompted calls for increased emphasis on the causes and consequences of phenotypic integration, or multivariate associations among traits, in global change research (Chirgwin et al., 2015; Etterson & Shaw, 2001; Hellmann & Pineda‐Krch, 2007; Teplitsky et al., 2014).

Characterizing patterns of phenotypic integration can reveal physiological trade‐offs that plants may experience in future carbon dioxide conditions (Tonsor & Scheiner, 2007; Ward & Kelly, 2004; Ward & Strain, 1999). For example, carbon supply and acquisition rates can influence water‐use efficiency; increased carbon supply may lead to decreased water loss because plants can reduce the density of stomata (i.e., microscopic pores on the surfaces of leaves that regulate gas exchange) without altering carbon acquisition rates (Eamus, 1991; Tonsor & Scheiner, 2007; Ward & Kelly, 2004). In future carbon dioxide conditions, this may enhance competition of some plants experiencing moderate drought (Woodward, Lake, & Quick, 2002; Lake & Woodward, 2008; although see Perry et al., 2013 for a different perspective). In addition, photosynthesis is typically accelerated in elevated carbon dioxide, resulting in increased carbohydrate production and biomass (Van der Kooij et al., 1999; Makino & Mae, 1999; Teng et al., 2006). However, without correlated increases in nitrogen, elevated carbon supply may reduce photosynthetic rate and generate trade‐offs among functional traits. This is due to the correlated acquisition and allocation of carbon and nitrogen, where increased carbon availability leads to increased nitrogen demand (Stitt & Krapp, 1999; Tonsor & Scheiner, 2007; Ward et al., 2000). Therefore, future carbon dioxide conditions may intensify existing trade‐offs in nitrogen‐deficient environments.

Characterizing phenotypic integration can also provide insights into evolutionary potential in rapidly changing environments. An established principle of quantitative genetics theory is that genetically correlated traits can influence rates of adaptation (Chirgwin et al., 2015; Etterson & Shaw, 2001; Goswami, Smaers, Soligo, & Polly, 2014; Hellmann & Pineda‐Krch, 2007; Teplitsky et al., 2014). Specifically, genetic correlations that are aligned with the direction of selection are predicted to promote, while antagonistic genetic correlations are predicted to constrain, adaptation (Agrawal & Stinchcombe, 2009; Chirgwin et al., 2015; Etterson & Shaw, 2001; Teplitsky et al., 2014). Crucially, genetic correlations may constrain responses to selection despite abundant heritable variation in univariate traits (Etterson & Shaw, 2001; Teplitsky et al., 2014; Walsh & Blows, 2009). Studies have shown that univariate and multivariate analyses can, as a result, produce different and sometimes contradictory estimates of evolutionary potential, underscoring the need for both approaches in global change studies (Chirgwin et al., 2015; Etterson & Shaw, 2001; Teplitsky et al., 2014; Walsh & Blows, 2009).

Lastly, multivariate plasticity may mitigate genetic constraints on adaptation in the short term, as it enables the expression of environment‐specific trait correlations without altering the underlying genetic architecture. A large body of work has shown that multivariate plasticity may influence patterns of selection and facilitate adaptation (Chevin, Lande, & Mace, 2010; Lind et al., 2015; Moczek et al., 2011; Murren, 2002, 2012; Pigliucci & Marlow, 2001; Pigliucci & Preston, 2004; Plaistow & Collin, 2014; Price, Qvarnstrom, & Irwin, 2003; Schlichting, 1989; Sgrò & Hoffmann, 2004; Wund, 2012). However, evidence also suggests that strong and nonlabile patterns of phenotypic integration may constrain univariate plasticity in plants: tighter integration among traits may reduce the range of variation expressed by individual traits (Gianoli & Palacio‐Lopez, 2009; Murren et al., 2015).

An important first step toward predicting short‐term responses to elevated carbon dioxide in plants is characterizing univariate and multivariate phenotypic plasticity within and among populations. Here, we explored population‐level differentiation in univariate and multivariate plasticity to elevated carbon dioxide in a set of six functional traits among six European accessions of *Arabidopsis thaliana*. We measured total fruit production (reproductive fitness), height (resource allocation to aboveground biomass), rosette diameter (resource allocation during the vegetative phase), biomass (resource allocation during the vegetative and reproductive phases), flowering time (an ecologically important phenological trait), and stomatal density (a functional trait that is crucial for regulating gas exchange). Previous studies have shown significant effects of carbon enrichment on these traits (Springer, Orozco, Kelly, & Ward, 2008; Springer & Ward, 2007; Ward & Kelly, 2004; Ward & Strain, 1999; Woodward et al., 2002), and significant variation in plasticity (i.e., genotype by environment interactions) in reproductive output (Ward & Strain, 1999), flowering time (Springer & Ward, 2007), and stomatal density (Woodward et al., 2002), but limited genetic variation in plasticity in traits associated with the production of biomass (Ward & Kelly, 2004; Ward & Strain, 1999). To our knowledge, multivariate associations in this set of six functional traits have not yet been addressed in the context of elevated carbon dioxide. We addressed the following questions: (a) How does elevated carbon dioxide affect trait means within and among populations? (b) Do populations harbor genetic variation for plasticity (i.e., genotype by environment interactions) to elevated carbon dioxide? (c) How does elevated carbon dioxide influence patterns of phenotypic integration and population‐level differentiation in the multivariate phenotype?

## 2 MATERIALS AND METHODS

### 2.1 Plant material and growth conditions

We used six natural accessions of *A. thaliana*, obtained from *The Arabidopsis Information Resource* (TAIR, http://www.arabidopsis.org): Canterbury, UK (Cnt‐1, accession 1679; 51.3 lat, 1.1 long); West Malling, UK (PHW‐13, accession 6013; 51.3 lat, 0.5 long); Sidmouth, UK (PHW‐23, accession 6023; 51.1 lat, −3.2 long); Coimbra, Portugal (Co, accession 3180; 40.2 lat, −8.4 long); Coimbra, Portugal (Co‐2, accession 6670, 40.1 lat, −8.3 long); and St. Maria da Feira, Portugal (Fei‐0, accession 22645; 40.9 lat, −8.5 long). We selected these accessions to explore differentiation in univariate and multivariate responses to elevated carbon dioxide within and among populations that originated from regions spanning a moderate latitudinal range (three accessions from the United Kingdom, 51.1–51.3 latitude; and three accessions from Portugal, 40.1–40.9 latitude). These regions differ in ecologically relevant climate variables that covary with latitude such as temperature, annual precipitation, and photoperiod (Table S1 for climatic data; Fick & Hijmans, 2017). Furthermore, these accessions are known to differ in their morphological characters, such as rosette shape and size at bolting, as well as flowering phenologies and reproductive characteristics.

Six growth chambers (1.2 m length × 0.6 m width × 0.8 m height) were used in this experiment.

Each chamber was fitted with a temperature, carbon dioxide, and humidity sensor (Li‐820 and Li‐840, LI‐COR Biosciences). Carbon dioxide was supplemented using solenoid valve‐controlled gas tank regulators (Titan Controls, Inc.) and maintained at 420 ppm in three low carbon dioxide chambers and at 840 ppm, the predicted concentration of atmospheric CO_2_ by year 2100 (IPCC, 2014), in three elevated carbon dioxide chambers. Environmental variables were monitored and controlled with Growtronix Software (Tronix Enterprises; growth chamber data logs are available upon request). Plants were grown in 10 × 10 × 8.5 cm pots with standard Pro‐Mix soil at 22°C in 16 hr of light per day (150 μmol m^−2^ s^−1^, measured at plant height; HO T12 linear fluorescent tubes were placed approximately 30 cm above plants).

### 2.2 Experimental design and measurements

To minimize maternal effects, 40 seeds per accession, derived from bulk‐propagated populations from the stock center, were randomly assigned to and grown in either the low (420 ppm) or high (840 ppm) carbon dioxide treatment (the maternal population). Six seeds were collected from each maternal plant and grown in the same carbon treatment as the parent (the experimental population). Prior to planting, seeds were imbibed with water and exposed to a five‐day cold treatment at 4°C in darkness. In total, the experiment consisted of 2 carbon dioxide treatments × 6 populations × 20 families × 6 replicates = 1,440 plants.

Measurements were taken on the following traits: total fruit production, a measure of reproductive fitness; height, a measure of resource allocation to aboveground biomass; rosette diameter (at bolting), a measure of resource allocation during the vegetative phase; dry weight (after drying at 60°C), a measure of resource allocation during the vegetative and reproductive phases; flowering time, an ecologically important phenological trait; and stomatal density, a functional trait that is crucial for regulating gas exchange and has been shown to respond to changes in carbon availability. Stomatal density was determined for three fully expanded rosette leaves per plant using the epidermal peel technique described in Ibata, Nagatani, and Mochizuki (2013). Stomata were then visualized using a Nikon Eclipse E200 compound microscope (Nikon Instruments, Inc.) with an integrated digital camera and counted by observing five separate fields of 0.16 mm^2^.

### 2.3 Data analysis

We used a split plot design where the effect of CO_2_ was tested separately from chamber (block) effects. To assess sources of variation for individual traits, we used multifactorial mixed model ANOVA (SPSS ver. 24; IBM Corp.) with the following sources of variation: population (genetic differentiation among accessions; fixed effect), family nested within population (genetic differentiation within populations; random effect), carbon dioxide (environmental treatment; fixed effect), population by treatment (variation for plasticity among populations), family by treatment (variation for plasticity within populations), chamber (experimental block). Log or square root transformations were performed to meet the assumptions of ANOVA, and *p*‐values were adjusted using a sequential Bonferroni correction when multiple comparisons were carried out. We used three distinct but related multivariate methods to explore variation in phenotypic integration among accessions within and between carbon dioxide treatments. First, we performed principal component analyses and determined trait loadings on leading eigenvectors. Second, we estimated eigenvalue variance to assess the strength of phenotypic integration (Armbruster, Pélabon, Bolstad, & Hansen, 2014; Pavlicev, Cheverud, & Wagner, 2009; Wagner, 1984). Stronger integration results in the concentration of variation in leading eigenvalues, which in turn leads to higher eigenvalue variance. In contrast, weaker integration produces similar eigenvalues with lower eigenvalue variance. Third, we performed pairwise comparisons of covariance matrices between unique population‐treatment groups using common principal components analysis (CPCA; Phillips & Arnold, 1999). CPCA is a multivariate statistical method used to detect structural similarities among covariance matrices. It is based on Flury's (1988) hierarchy and has been adapted to quantitative genetic data by Phillips and Arnold (1999). In contrast to approaches that test for only equality, CPCA tests a series of nested models of structural similarity between covariance matrices. The model hierarchy begins with the assumption of unrelated covariance structure. This is followed by a series of increasingly constrained models that include partial CPCs, full CPCs, proportionality, and equality. This approach enables the detection of subtle differences in covariance structure. Using the “jump‐up” approach of CPCA (Phillips & Arnold, 1999), we indicated the best‐fitting model for each comparison as the one below the point in the hierarchy where CPCA found statistically significant differences between matrices (i.e., the best model in the hierarchy is the one below the rejected model).

## 3 RESULTS

Analysis of variance revealed significant population‐level variation in all traits and significant within‐population, family‐level variation in only one trait (fruit number; Table 1). Elevated carbon dioxide had significant effects on trait means (Table 1). Stomatal density decreased by 11.28%, and the other five traits increased by 10.87%–43.61% (Table 2). Only one trait (flowering time; Table 1) showed significant variation in plasticity among accessions. In contrast, we did not detect significant family‐level variation in plasticity (i.e., GxE interaction within populations) in any trait (Table 1).

**Table 1:** *F‐*values showing the effects of elevated carbon dioxide, population, family, and their interaction on six traits in *Arabidopsis thaliana*

	Flowering time	Weight	Rosette diameter	Height	Fruit number	Stomatal density
Population (*df* = 5)	3.41**	4.23**	4.72***	3.94**	9.16***	4.94***
Family (population) (*df* = 19)	0.02	0.98	0.74	1.40	1.71***	1.42
CO_2_ (*df* = 1)	8.72**	19.48***	13.92**	32.64***	9.57**	12.73**
Population × CO_2_ (*df* = 5)	5.86***	1.81	2.17	1.65	1.93	1.38
Family × CO_2_ (*df* = 19)	0.77	0.91	0.18	1.38	1.17	0.79

a *p < 0.05, **p < 0.01, ***p < 0.001; *df*, degrees of freedom.

**Table 2:** Means for six *Arabidopsis thaliana* traits in ambient and elevated carbon dioxide treatments

	420 ppm	840 ppm
Flowering time (days)	37.14 (±0.49)	41.18 (±0.38)
Weight (mg)	29.31 (±1.12)	37.53 (±1.17)
Rosette diameter (cm)	3.21 (±0.10)	4.65 (±0.19)
Height (cm)	19.15 (±0.29)	24.02 (±0.27)
Stomatal density (mm^−2^)	235.43 (±2.08)	209.87 (±2.61)
Fruit number	44.67 (±1.51)	61.43 (±1.77)

Note

Data shown are trait means ± 1 *SE*.

Correlation analysis showed 14 out of 15 significant correlations in the 420 ppm treatment and 12 out of 15 significant correlations in the 840 ppm treatment (Table 3). We found three significant negative correlations in 420 ppm treatment and one significant negative correlation in 840 ppm treatment. The correlation between rosette diameter and stomatal density changed from weak negative (*r* ^2^ = −0.08; *p* < 0.05) in 420 ppm to moderately strong positive (*r* ^2^ = 0.49; *p* < 0.05) in 840 ppm (Table 3). The correlation between weight and flowering time changed from positive (*r* ^2^ = 0.22; *p* < 0.05) in 420 ppm to negative (*r* ^2^ = −0.34; *p* < 0.05) in 840 ppm (Table 3).

**Table 3:** Correlations among six traits of *Arabidopsis thaliana* in two carbon dioxide treatments (420 ppm = below diagonal; 840 ppm = above diagonal)

	Flowering time	Weight	Rosette diameter	Height	Fruit number	Stomatal density
Flowering time	1	**−0.34**	−0.03	**0.26**	−0.04	**0.38**
Weight	**0.22**	1	**0.42**	**0.23**	**0.14**	−0.05
Rosette diameter	**−0.11**	**0.32**	1	**0.43**	**0.28**	**0.49**
Height	**0.23**	**0.17**	**0.13**	1	**0.39**	**0.36**
Fruit number	**−0.17**	−0.06	**0.25**	**0.69**	1	**0.19**
Stomatal density	**0.42**	**0.25**	**−0.08**	**0.16**	**0.17**	1

Note

Data shown are Pearson correlation coefficients. Boldface indicates significant correlation (*p* < 0.05).

**Figure 1:**
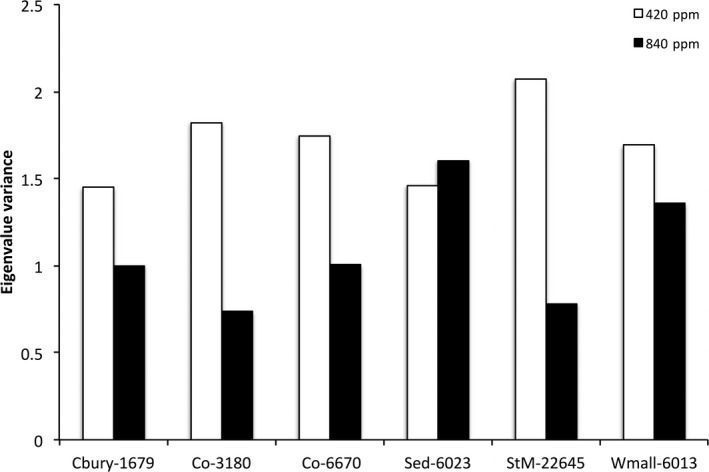
The effects of elevated carbon dioxide on eigenvalue variance, a measure of the strength of phenotypic integration, in six populations of *Arabidopsis thaliana*. Cbury‐1679: Canterbury, UK; Co‐3180: Coimbra, PO; Co‐6670: Coimbra, PO; Sed‐6023: Sidmouth, UK; StM‐22645: St. Maria da Feira, PO; Wmall‐6013: West Malling, UK

**Figure 2:**
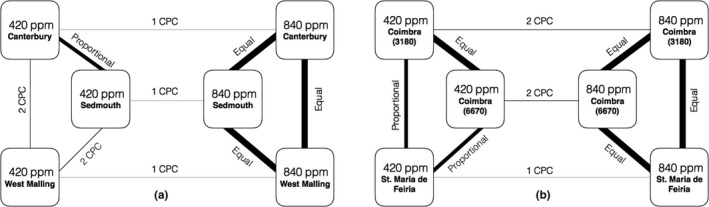
Summary of common principal components analyses (CPCA) showing population‐level variation in phenotypic integration within and across carbon dioxide treatments in (A) three United Kingdom (51.1–51.3 latitude) and (B) three Portuguese accessions of *Arabidopsis thaliana* (40.1–40.9 latitude). Lines represent pairwise CPCA comparisons of covariance matrices. Line thickness corresponds to CPCA result: unrelated matrices (no line), 1–5 CPCs, matrix proportionality, and matrix equality (thickest line)

Elevated carbon dioxide resulted in decreased eigenvalue variance, a measure of the strength of phenotypic integration, in all accessions except Sidmouth, UK (Figure 1). Principal components analyses revealed that the first two components accounted for 62.63% and 57.24% of the variance in low and high carbon treatments, respectively (Table 4). Trait loadings showed that in 420 ppm treatment, principal component 1 (PC1) was influenced mainly by variation in weight, rosette diameter, height, and stomatal density, and PC2 was influenced mainly by flowering time and fruit number (Table 4). In contrast, in 840 ppm treatment, fruit number and weight loaded more heavily onto PC1 and PC2, respectively (Table 4). Common principal components analysis (CPCA) revealed variable amount of population‐level differentiation in the multivariate phenotype in low carbon dioxide treatment and no detectable population‐level differentiation in high carbon dioxide treatment (Figure 2). Furthermore, we found very little common structuring in pairwise comparisons of trait covariance matrices across carbon dioxide treatments. Specifically, among UK accessions grown in 420 ppm carbon dioxide, we found two CPCs in the Canterbury‐West Malling and Sidmouth‐West Malling comparisons, and proportional matrices in the Canterbury‐Sidmouth comparison (Figure 2). Among Portuguese accessions grown in 420 ppm carbon dioxide, we found equal matrices between the two Coimbra accessions and proportional matrices between each of the Coimbra accessions and the St. Maria da Feira accession. In contrast, in the 840 ppm carbon treatment, all pairwise CPCA comparisons in both UK and PO accessions resulted in equal matrices (i.e., we did not detect differentiation in covariance matrices among accessions grown at elevated carbon dioxide), suggesting that increased carbon availability resulted in similar and relatively weak trait covariances. Furthermore, we found only one or two CPCs in pairwise comparisons between trait covariance matrices of accessions grown in low and high carbon dioxide treatments (Figure 2), indicating that elevated carbon dioxide altered trait covariance structure.

**Table 4:** Eigenvalues and eigenvectors in low and elevated carbon dioxide treatments

CO_2_ treatment	420 ppm		840 ppm	
Principal component	1	2	1	2
Eigenvalue	2.23	1.53	1.96	1.55
% variance explained	37.11	25.52	32.43	25.81
Eigenvectors				
Flowering time	0.23	**0.75**	0.38	**0.84**
Weight	**0.39**	0.35	0.46	**−0.75**
Rosette diameter	**0.80**	−0.37	**0.39**	−0.23
Height	**0.78**	−0.24	**0.83**	0.08
Fruit number	0.57	**−0.59**	**0.70**	−0.16
Stomatal density	**0.68**	0.56	**0.53**	0.45

Note

Boldface indicates the principal component with which the trait is more strongly associated.

## 4 DISCUSSION

The unprecedented rise of atmospheric carbon dioxide—a key driver of global climate change—represents a significant ecological challenge for plants (Jump & Penuelas, 2005; Leakey & Lau, 2012; Terrer et al., 2017; Ward & Kelly, 2004). Characterizing patterns of phenotypic integration and plasticity to elevated carbon dioxide can provide insights into initial responses to global change. Here, we investigated population‐level differentiation in univariate and multivariate plasticity to elevated carbon dioxide in a set of six traits among six European accessions of *A. thaliana*. We found that elevated carbon dioxide (a) induced significant plastic responses in both univariate and multivariate phenotypes, but we did not find significant variation in plasticity (genotype by environment interactions) within or among accessions; (b) altered patterns of trait covariance, reduced the strength of phenotypic integration, and decreased accession‐level differentiation in trait covariance structure.

### 4.1 Univariate plasticity to elevated carbon dioxide

We detected significant population‐level variation in all traits and significant family‐level variation in only fruit production. These patterns are consistent with previously documented quantitative variation in *A. thaliana* (Koornneef, Alonso‐Blanco, & Vreugdenhil, 2004; Kuittinen, Mattila, & Savolainen, 1997; Pigliucci, 2002), a mostly self‐fertilizing species that is expected to harbor more genetic variation among than within populations. In addition, while elevated carbon dioxide‐induced plastic responses in all traits, we did not detect significant variation in plasticity (i.e., genotype by environment interaction, or G × E) in any trait except flowering time. Plasticity to elevated carbon dioxide in flowering time has been found to vary among *A. thaliana* ecotypes (reviewed in Springer & Ward, 2007). For example, Springer and Ward (2007) found significant variation in flowering time plasticity to elevated CO_2_ among 10 widely distributed *Arabidopsis* ecotypes. Interestingly, all possible responses were observed, including unaltered, accelerated, and delayed flowering. Since the ecotypes originated from widespread regions characterized by large differences in environmental parameters (temperature, water availability, and photoperiod), the authors suggested that divergent local selective pressures on integrated life history traits may have influenced variation in flowering time responses to elevated CO_2_. While genetic variation in flowering time plasticity may point to divergent selection operating on the reaction norm (Springer & Ward, 2007; Ward et al., 2000), this pattern is also consistent with the idea that at least some of the response variation is conditionally neutral (Ghalambor, McKay, Carroll, & Reznick, 2007; Marais, Hernandez, & Juenger, 2013; Schlichting, 2008).

Stomatal density is sensitive to a wide range of CO_2_ concentrations (including preindustrial levels), and responses in this trait have been shown to vary in a genotype‐dependent manner in *Arabidopsis* (Lake & Woodward, 2008; Ward & Kelly, 2004; Ward & Strain, 1999; Woodward et al., 2002). We found an approximate 11% decrease in stomatal density in elevated carbon dioxide, which is consistent with some previous findings for this trait under carbon enrichment (Lake & Woodward, 2008; Woodward et al., 2002). This result supports the notion that plants in carbon‐rich environments can afford to reduce stomatal densities while maintaining carbon intake and water‐use efficiency (Eamus, 1991; Tonsor & Scheiner, 2007). Our experiment was not designed to measure photosynthetic rates or water‐use efficiency, but future empirical work may consider quantifying these traits along with stomatal densities in a multivariate context. We suggest that the patterns of integration among these traits may likely be altered by elevated carbon dioxide, and trade‐offs among photosynthetic rate, biomass, and stomatal densities may arise, especially in nitrogen‐deficient environments due to shared resource allocation (see *Introduction*; Stitt & Krapp, 1999).

In addition to significant variation in flowering time responses described above, based on previous work we expected to find significant genetic variation in plasticity of fitness (Ward & Strain, 1999), stomatal density (Woodward et al., 2002), and limited genetic variation in the plasticity of traits associated with the production of biomass (rosette diameter, height, and biomass; Ward & Strain, 1999; Ward & Kelly, 2004). However, our results are, overall, in line with work showing significant plastic responses to elevated CO_2_ in morphological, physiological, phenological, and fitness‐related traits (reviewed in Leakey & Lau, 2012; Ward & Kelly, 2004; Ward & Strain, 1999; Wieneke, Prati, Brandl, Stöcklin, & Auge, 2004), but little or no genetic variation in phenotypic plasticity to elevated CO_2_ (Lau, Shaw, Reich, Shaw, & Tiffin, 2007; Leakey & Lau, 2012; Roumet, Laurent, Canivenc, & Roy, 2002; Volk & Körner, 2001; although see Lindroth, Roth, & Nordheim, 2001). Specifically, among studies focused on variation in plasticity to elevated CO_2_ in biomass and fitness (seed number, fruit number, seed weight, or relative growth rate), less than a third have found statistically significant GxE in these traits: Lau et al. (2007) and Roumet et al. (2002) report 7/21 and 11/39 studies, respectively, that have found significant GxE in such traits.

### 4.2 Multivariate plasticity to elevated carbon dioxide

Multivariate analyses showed evidence of multivariate plasticity to elevated carbon dioxide. Common principal components analysis (CPCA) detected little common structuring (1–2 CPCs) between trait covariance matrices between carbon dioxide treatments, suggesting that elevated carbon dioxide‐induced shifts in the patterns of phenotypic integration (Figure 1). Principal components analysis indicated that these shifts involved changes in the loadings of biomass, rosette diameter, and reproductive output (fruit number) on the leading eigenvectors (Table 4). Furthermore, analysis of bivariate trait correlations and eigenvalue variance revealed fewer negative correlations and weaker integration in elevated carbon treatment (Figure 1; Tables 1 and 2). These results support both theoretical and empirical work centered on resource acquisition and allocation in plants. Studies have found that environmental stress, such as resource limitation, intensifies trade‐offs (i.e., negative correlations) and increases the strength of integration among functional traits (Gianoli & Palacio‐Lopez, 2009; Murren, 2012; Pigliucci & Kolodynska, 2002; Pigliucci & Marlow, 2001; Valladares, Gianoli, & Gómez, 2007). However, when resources (e.g., carbon supply) are less limiting, the intensity of trade‐offs and the strength of phenotypic integration tend to decrease (Bidart‐Bouzat, Portnoy, DeLucia, & Paige, 2004; Murren, 2012; Tonsor & Scheiner, 2007). Furthermore, shifts toward weaker phenotypic integration are predicted to facilitate independent variation in traits and promote evolutionary potential in univariate dimensions. In turn, changes in phenotypic integration may alter patterns of selection and evolutionary outcomes in *A. thaliana*. Evidence suggests that elevated carbon dioxide can, in fact, relax competition‐mediated selection (Lau et al., 2014) and influence the strength of selection on carbon assimilation in *Arabidopsis* (Tonsor & Scheiner, 2007).

We found some degree of population‐level differentiation in phenotypic integration in low carbon dioxide treatment in both the UK and PO regions, as indicated by pairwise CPCAs of trait covariance matrices (Figure 2). It is well known that *A. thaliana* is a highly selfing species and ecotypes are differentiated in their life history characteristics (Koornneef et al., 2004; Pigliucci, 2002). Since similar patterns of multivariate responses arise as a result of shared developmental‐genetic mechanisms, we speculate that divergent patterns of phenotypic integration among geographically widespread accessions may reflect natural variation in genetic architecture (Bidart‐Bouzat et al., 2004). A fundamental question concerning the genotype–phenotype map (sensu Wagner & Altenberg, 1996; Wagner & Zhang, 2011) is how variation in genetic architecture shapes phenotypic integration. Addressing this question using a combination of quantitative genetics, molecular genetics, and genomics approaches could deepen our understanding of adaptation to climate change in plants.

In contrast to low carbon dioxide treatment, we did not detect differentiation among trait covariance matrices among accessions in elevated carbon treatment in either the UK or PO regions (Figure 2). Therefore, different lines of evidence from this study—Pearson correlations, eigen variance analyses, PCA, and CPCA—support the conclusion that elevated carbon dioxide induced shifts in the patterns of integration and trait covariances among accessions became similarly weak. The erosion of variation in phenotypic integration in elevated carbon treatment may have significant implications for climate‐driven evolution in *A. thaliana*. Similar patterns of trait covariance are predicted to impose similar biases on evolutionary trajectories. Therefore, our data suggest that geographically widespread ecotypes of *A. thaliana* will likely experience similar patterns of relaxed constraint on adaptation in future carbon dioxide conditions.

Although we used a unique set of statistical approaches (correlation analysis, eigenvalue variance analysis, PCA, CPCA), our finding that trait integration is sensitive to changes in carbon availability is in general agreement with previous experiments that used principal components analyses (Bidart‐Bouzat et al., 2004) and structural equation modeling (Tonsor & Scheiner, 2007) to characterize the effects of carbon enrichment on phenotypic integration in *A. thaliana*. Therefore, this finding appears to be robust to both variation in traits measured and differences among multivariate techniques used to analyze patterns and magnitudes of integration. In addition, our predictions that elevated carbon dioxide will likely mitigate trade‐offs and relax constraints on adaptation are in line with a previous study that also found significant shifts in patterns of integration, as well as partial decoupling between traits (carbon assimilation and transpiration rates) and weaker selection on carbon assimilation in *A. thaliana* accessions grown across a CO_2_ gradient (Tonsor & Scheiner, 2007). Finally, our findings are overall consistent with the broader idea, supported by a large body of work, that rapid changes in the environment can have significant impacts on phenotypic integration (Bidart‐Bouzat et al., 2004; Chevin et al., 2010; Lind et al., 2015; Marroig & Cheverud, 2001; Moczek et al., 2011; Murren, 2002, 2012; Pigliucci & Preston, 2004; Plaistow & Collin, 2014; Price et al., 2003; Schlichting, 1989; Sgrò & Hoffmann, 2004; Wund, 2012).

Additional work would be needed to test the robustness of these results in more complex biotic environments. Previous work has shown that the presence of additional ecological variables, such as herbivores and competitors, can alter the predicted evolutionary effects of elevated carbon dioxide on plants (Bidart‐Bouzat, Mithen, & Berenbaum, 2005; Bidart‐Bouzat et al., 2004; Lau et al., 2014). Future studies that combine ecological complexity with population‐level multivariate analyses of phenotypic plasticity and integration could deepen our understanding of evolutionary potential of plants in the face of climate change.

## CONFLICT OF INTEREST

None declared.

## AUTHOR CONTRIBUTIONS

M.J. designed the study, collected data, analyzed data, and wrote the manuscript. B.C. collected data and analyzed data.

## Data Availability

Data are available on Dryad: https://doi.org/10.5061/dryad.c0k235b.
