# Supplementary material for: Patterns of univariate and multivariate plasticity to elevated carbon dioxide in six European populations of Arabidopsis thaliana
Source: Ecol Evol. 2019 May 8;9(10):5906–15. doi: 10.1002/ece3.5173 (PMC6540656; doi:10.1002/ece3.5173)
Supplement: Supplementary file 1 [file ECE3-9-5906-s001.docx]

Supplementary Table

Table S1. Climatic data for regions of origin for each *A. thaliana* accession used in this study (NOAA; Fick and Hijmas 2017).

| Accession | | Region | Lat | Long | Daytime high temperature (°C) (January/July) | Total annual precipitation (mm) | Photoperiod (hrs) (January/July) |
| --- | --- | --- | --- | --- | --- | --- | --- |
| Cnt-1 | CS1679 | Canterbury, UK | 51.3 | 1.1 | 7/21 | 614 | 8.5/16.5 |
| PHW-13 | CS6013 | West Malling, Kent, UK | 51.3 | 0.6 | 8/23 | 639 | 8.5/16.5 |
| PHW-23 | N6023 | Sidmouth, Devon, UK | 51.1 | -3.2 | 9/21 | 832 | 8.5/16.5 |
| Co | CS3180 | Coimbra, PO | 40.2 | -8.4 | 15/29 | 922 | 9.5/15 |
| Co-2 | CS6670 | Coimbra, PO | 40.2 | -8.3 | 15/29 | 922 | 9.5/15 |
| Fei-0 | CS22645 | St. Maria da Feira, PO | 40.9 | -8.5 | 14/25 | 998 | 9.5/15 |
